# Early Clinical and Economic Outcomes for the VELYS Robotic-Assisted Solution Compared with Manual Instrumentation for Total Knee Arthroplasty

**DOI:** 10.1055/a-2343-2444

**Published:** 2024-06-28

**Authors:** Philip Huang, Michael Cross, Anshu Gupta, Dhara Intwala, Jill Ruppenkamp, Daniel Hoeffel

**Affiliations:** 1 OrthoIndy, Indianapolis, Indiana; 2 Epidemiology and Real-World Data Sciences, Johnson and Johnson MedTech, New Brunswick, New Jersey; 3 DePuy Synthes Digital, Robotics and Emerging Channels, Raynham, Massachusetts; 4 DePuy Synthes, Medical Affairs, Palm Beach Gardens, Florida

**Keywords:** robotic surgery, total knee arthroplasty, VELYS, VRAS, real world data

## Abstract

Robotic-assisted total knee arthroplasty (TKA) has been developed to improve functional outcomes after TKA by increasing surgical precision of bone cuts and soft tissue balancing, thereby reducing outliers. The DePuy Synthes VELYS robotic-assisted solution (VRAS) is one of the latest entrants in the robotic TKA market. Currently, there is limited evidence investigating early patient and economic outcomes associated with the use of VRAS. The Premier Healthcare Database was analyzed to identify patients undergoing manual TKA with any implant system compared with a cohort of robotic-assisted TKAs using VRAS between September 1, 2021 and February 28, 2023. The primary outcome was all-cause and knee-related all-setting revisits within 90-day post-TKA. Secondary outcomes included number of inpatient revisits (readmission), operating room time, discharge status, and hospital costs. Baseline covariate differences between the two cohorts were balanced using fine stratification methodology and analyzed using generalized linear models. The cohorts included 866 VRAS and 128,643 manual TKAs that had 90-day follow-up data. The rates of both all-cause and knee-related all-setting follow-up visits (revisits) were significantly lower in the VRAS TKA cohort compared with the manual TKA cohort (13.86 vs. 17.19%; mean difference [MD]: −3.34 [95% confidence interval: −5.65 to −1.03] and 2.66 vs. 4.81%; MD: −2.15 [−3.23 to −1.08], respectively, *p* -value < 0.01) at 90-day follow-up. The incidence of knee-related inpatient readmission was also significantly lower (53%) for VRAS compared with manual TKA. There was no significant difference between total cost of care at 90-day follow-up between VRAS and manual TKA cases. On average, the operating room time was higher for VRAS compared with manual TKA (138 vs. 134 minutes). In addition, the discharge status and revision rates were similar between the cohorts. The use of VRAS for TKA is associated with lower follow-up visits and knee-related readmission rates in the first 90-day postoperatively. The total hospital cost was similar for both VRAS and manual TKA cohort while not accounting for the purchase of the robot.

Total knee arthroplasty (TKA) is a well-established and cost-effective procedure for the treatment of end-stage knee osteoarthritis. To further improve surgical outcomes, robotic-assisted solutions have been developed to increase surgical precision and reduce surgical variability. Robotic technology in TKA has been shown to improve patient outcomes, especially range of motion, patient satisfaction, and facilitate a shorter recovery time. 1 2 3 4 5 6 7 Current literature also suggests that use of robotic-assisted TKA can reduce soft tissue trauma leading to decreased pain and expedited recovery. 8 9 However, some studies have suggested that the benefits of robotic-assisted TKA may be only apparent in the early postoperative period 10 11 and have highlighted concerns regarding the learning curve 12 13 and increased costs associated with the use of robotic surgery. 14 15

The DePuy Synthes VELYS robotic-assisted solution (VRAS) is one of the latest entrants in the rapidly evolving field of robotic technology for TKA. VRAS is an imageless system designed to eliminate the need for preoperative CT scans, which can lower preoperative preparation time, cost, and radiation exposure. It is only compatible with the ATTUNE Knee System (DePuy Synthes), a widely used knee implant, and has the ability to facilitate precise, accurate, and informed decision-making during surgery. 16 Early results from recent studies have shown promising results for the use of VRAS in TKA. 17 18

Most of the current literature is focused on evaluating robotic technology for TKA as a class or related to one of the more established robotic systems. Current VRAS-specific evidence is generally focused around single sites 17 18 or cadaveric studies. 19 20 This retrospective comparative study is designed to evaluate early postoperative clinical and economic outcomes with the use of VRAS in TKA compared with a large cohort of manual TKAs, utilizing a large hospital billing database.

## Methods

### Data Source

Data from the Premier Healthcare Database were used to identify patients undergoing manual TKA with any implant system compared with a cohort of robotic-assisted TKA using VRAS. The Premier Healthcare Database is nationally representative and encompasses extensive clinical coding information, including diagnoses, procedures, and hospital-administered medications. 21 It draws data from more than 1,000 hospitals and healthcare systems, covering over 20% of all hospital admissions in the United States. Additionally, the database includes a chargemaster, which includes device-specific details. The database was reviewed by the New England Institutional Review Board (IRB) and was determined to be exempt from IRB approval.

### Study Population

Patients with a Current Procedural Terminology code or International Classification of Diseases, Tenth Revision (ICD-10) code indicative of primary TKA from September 1, 2021 to February 28, 2023 were included in the study. The start date of data collection was based on the first available VRAS TKA in the database. The date of the admission for the TKA procedure was defined as the index date. Utilizing ICD-10 data and demographic information, patients with any of the following criteria were excluded from the study: age < 18 years, diagnosis for aseptic loosening, infection, osteomyelitis, knee fracture at the time of index, or had a partial-knee procedure. Additionally, the following patients were excluded from 90-day follow-up analysis: patients that underwent a second primary procedure within 90 days of index or had continuous enrollment for less than 90-day postindex.

### Variables

Patient demographics included age, gender, race, marital status, payer type, procedure setting (inpatient or outpatient), smoking status, and comorbidities. Baseline comorbidities were assessed using the Elixhauser Comorbidity Index and Functional Comorbidity Index (FCI). The overall Elixhauser score reflects the overall comorbidity level by assessing 31 dimensions related to chronic diseases. Additionally, this score has demonstrated an association with the risk of mortality and healthcare resource utilization. 22 23 The FCI includes 18 medical conditions and holds relevance in orthopaedic care as it was created as a measure of patient functional capacity. 24 25 Provider characteristics included hospital bed size, annual volume of TKA procedures (per hospital/physician), geographic location, hospital location (urban or rural), and teaching status. Procedural characteristics included admission year and fixation type (cemented or uncemented).

### Outcomes

The primary outcome was all-setting follow-up visits (revisits) within 90-day post-TKA. Secondary outcomes included readmission rates within 90-day, operating room time, discharge status (home vs. skilled nursing facility), and hospital costs including index and 90-day total cost of care (index + follow-up cost). Hospital cost at index was further subcategorized into supply and operation room cost.

### Data Analysis

All variables and outcomes listed above were analyzed using standard descriptive statistics. Continuous variables were presented in terms of means and standard deviations (along with 95% confidence intervals [CIs]), binary outcomes were presented as proportions with 95% CIs. To control the differences between the VRAS and manual TKA cohorts' fine stratification and weighing (FSW) methodology was used. 26 27 A total of 200 strata were created, and no individual patient information was discarded using this method. A love-plot was generated to show changes in standardized mean difference (SMD) between pre- and postbalancing of the covariates. Absolute SMD of 0.2 was used to assess good covariate balance. 28 Subsequently, weighted generalized linear regression models were utilized to calculate the adjusted effect of the exposure after stratification. All costs were inflated to 2023 U.S. dollars using the Bureau of Labor Statistics consumer price index. 29

## Results

Table 1 provides the distribution of the population for each cohort. A total of 1,180 VRAS TKA and 161,866 manual TKA cases were included in the study, with 866 VRAS and 128,643 manual TKA cases having 90-day follow-up data.

**Table 1:** Patient attrition using the inclusion and exclusion criteria

	Description	VRAS TKA	Manual TKA
1	Include patients with primary TKA surgery from Q4 2015 to April 2023	1,814	1,388,591
2	Include patients with data Publish_type = CP	1,665	1,264,423
3	Excluding patients (and all of their episodes) with at least 2 or more pat_keys with same admission dates	1,665	1,263,860
4	Take Index admission episode	1,413	1,097,711
5	Include patients with age ≥ 18	1,413	1,097,584
6	Include elective patients only	1,343	1,036,615
7	Exclude patients with fracture of knee at index	1,340	1,032,147
8	Exclude patients with diagnosis of aseptic loosening at index	1,336	1,009,651
9	Exclude patients with cancer diagnosis at index	1,332	1,005,842
10	Exclude patients with diagnosis of infection/osteomyelitis at index	1,330	997,841
11	Exclude patients without unknown gender	1,330	997,670
12	Exclude patients 0 costs at index	1,330	992,895
13	Exclude partial knee patients	1,330	981,711
14	Exclude manual TKA patients before September 1, 2021 (VELYS data availability)	1,330	161,866
15	Exclude VRAS patients that have indication of any other robotic technology usage	1,180	161,866
A a	Exclude patients that have less than 90-day follow-up data	885	133,892
B a	Exclude patients that have bilateral procedures within 90 days	866	128,643

Abbreviations: TKA, total knee arthroplasty; VRAS, VELYS robotic-assisted solution.

a A and B criteria only applied for 90-day follow-up analysis.

### Patient and Provider Baseline Characteristics

The patient and provider baseline characteristics of the study cohorts are presented in Tables 2 and 3 , respectively. The VRAS and manual TKA patients exhibited overall similarity (SMD < 0.2) in terms of demographics and comorbidities. The majority of patients were married, Caucasian women of similar age, and with Medicare as the primary payor. Approximately half of the patients in both cohorts had one to two comorbidities. The only significant baseline difference observed was in patients with existing knee pain indication, where the VRAS cohort (31%) had a higher prevalence compared with the manual TKA cohort (8%), with an SMD of 0.62. The majority of the cases were outpatient cases with almost 97% of VRAS TKA and 90% of manual TKA cases being outpatient.

**Table 2:** Patient characteristics of patients undergoing total knee arthroplasty using either manual approach or VELYS robotic-assisted solution, before and after fine stratification and weighting

Variable	Prefine stratification			Postfine stratification		
	Manual	VRAS	SMD	Manual	VRAS	SMD
*N*	128,643	866		128,643	866	
Age, mean (SD)	68.00 (9.20)	67.73 (8.94)	0.03	67.59 (9.17)	67.73 (8.94)	0.015
Age category (%)			0.057			0.028
18–34	0.08	0		0	0	
35–44	0.85	0.92		1.09	0.92	
45–54	7.07	7.62		7.46	7.62	
55–64	25.39	25.29		25.15	25.29	
65–74	41.49	42.61		43.52	42.61	
75 and above	25.11	23.56		22.78	23.56	
Gender: men (%)	38.98	38.8	0.004	36.47	38.8	0.048
Marital status (%)			0.129			0.045
Married	60.58	66.74		65.89	66.74	
Single	35.75	30.37		31.8	30.37	
Other	3.66	2.89		2.31	2.89	
Race (%)			0.137			0.08
Asian	1.47	1.62		1.66	1.62	
Black	9.71	7.62		8.72	7.62	
Other	6.04	3.7		2.46	3.7	
White	82.78	87.07		87.15	87.07	
Payer (%)			0.069			0.065
Commercial	27.04	30.14		28.64	30.14	
Medicaid	4.63	4.39		4.10	4.39	
Medicare	64.09	61.32		61.86	61.32	
Other	4.24	4.16		5.40	4.16	
Functional Comorbidity Index, mean (SD)	3.23 (1.62)	3.13 (1.65)	0.059	2.98 (1.78)	3.13 (1.65)	0.088
Elixhauser Comorbidity Index, mean (SD)	2.03 (1.57)	1.83 (1.54)	0.128	1.77 (1.62)	1.83 (1.54)	0.041
Elixhauser categories (%)			0.184			0.094
No comorbidities	16.86	24.02		27.9	24.02	
1–2	49.46	45.15		41.58	45.15	
3–4	26.47	25.17		24.71	25.17	
5 or greater	7.21	5.66		5.81	5.66	
Additional condition (%)						
Knee pain	7.71	31.18	0.621	36.19	31.18	0.106
Smoking	29.62	34.18	0.098	32.3	34.18	0.04
Arthritis	97.93	99.65	0.158	99.48	99.65	0.02
COPD	6.12	4.97	0.051	4.47	4.97	0.02
Heart failure	63.53	55.77	0.159	52.9	55.77	0.06
Diabetes	21.73	17.09	0.118	15.71	17.09	0.04
Obesity	32.14	27.14	0.11	28.58	27.14	0.03

Abbreviations: COPD, chronic obstructive pulmonary disease; SD, standard deviation; SMD, standardized mean difference; TKA, total knee arthroplasty; VRAS, VELYS robotic-assisted solution.

**Table 3:** Provider characteristics of patients undergoing total knee arthroplasty using either manual approach or VELYS robotic-assisted solution, before and after fine stratification and weighting

Variable	Prefine stratification			Postfine stratification		
	Manual	VRAS	SMD	Manual	VRAS	SMD
Number of patients	128,643	866		128,643	866	
Urban hospital (vs. rural) (%)	86.56	96.88	0.381	95.42	96.88	0.076
Region (%)			1.273			0.108
Midwest	30.18	10.39		12.59	10.39	
Northeast	13.32	61.66		57.77	61.66	
South	42.8	27.94		29.43	27.94	
West	13.69	0		0.2	0	
Hospital bed size (%)			0.886			0.227
000–099	12.94	7.85		6.99	7.85	
100–199	19.6	28.64		27.47	28.64	
200–299	19.61	10.85		15.01	10.85	
300–399	17	28.52		33.26	28.52	
400–499	10.96	24.02		16.85	24.02	
500 and above	19.9	0.12		0.42	0.12	
Teaching hospital (vs. community) (%)	41.57	81.76	0.908	75.14	81.76	0.161
Annual provider volume (%)			0.423			0.103
000–138	18.07	25.98		26.69	25.98	
139–313	27.82	27.02		26.5	27.02	
314–576	27.57	12.12		15.22	12.12	
Above 576	26.53	34.87		31.59	34.87	
Annual physician volume (%)			0.607			0.2
0–20	17.54	6.58		8.71	6.58	
21–50	22.92	11.09		16.91	11.09	
51–100	25.02	21.13		20.41	21.13	
Above 100	34.53	61.2		53.97	61.2	
Cemented fixation (vs. uncemented) (%)	2.09	1.62	0.035	2.01	1.62	0.03
Inpatient procedures (%)	10.21	2.77	0.305	2.73	2.77	0.002

Abbreviations: SMD, standardized mean difference; TKA, total knee arthroplasty; VRAS, VELYS robotic-assisted solution.

There was a significant difference (SMD > 0.2) in some of the baseline provider characteristics between the VRAS and manual TKA cohorts. Most patients in both cohorts were admitted to urban hospitals, 97% of VRAS and 87% of manual TKA cases, with SMD of 0.38. However, there was a significant difference in the hospital location (SMD: 1.27) with VRAS cases predominantly performed in Northeast (62%) and a majority of manual cases being performed in the South (43%). In the VRAS cohort, most hospitals (82%) were teaching hospitals, whereas in the manual cohort they were primarily community hospitals (58%), with an SMD of 0.91. The difference in hospital bed size was significantly different, with an SMD of 0.89. Annual provider and physician TKA volumes exhibited significant differences, with SMDs of 0.42 and 0.61, respectively.

### Patient and Provider Postcovariate Balancing Characteristics and Outcome Results

**Fig. 1:**
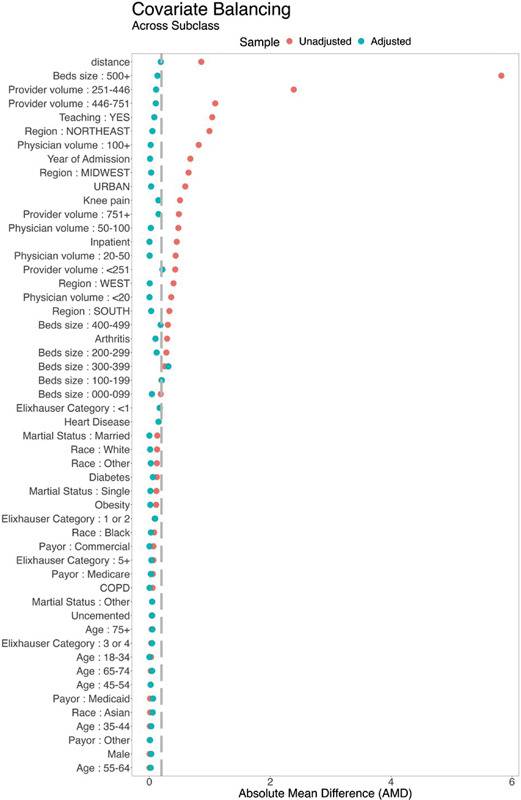
Covariate balance before and after fine stratification—90-day follow-up.

Tables 2 and 3 display the patient and provider characteristics following covariate balancing using the FSW method. In general, a satisfactory balance was achieved, with most SMDs below 0.20 ( Fig. 1 ). Patient characteristics stayed consistent, all having SMDs below 0.2. The provider characteristics were well-balanced across cohorts, with the majority having SMDs below 0.20. The one exception was hospital bed size, which had slightly higher SMD of 0.23, reduced from 0.89. This variable was included in the regression analysis to account for any remaining imbalance. Patients comorbidities were balanced across both cohorts and details are included in supplemental Table A and B (available online).

### Primary Outcomes

The primary outcomes of the study populations within 90-day follow-up are presented in Table 4 . The rates of both all-cause and knee-related all-setting follow-up visits (revisits) were significantly lower in the VRAS TKA cohort compared with the manual TKA cohort (13.86 vs. 17.19%; mean difference [MD]: −3.34 [95% CI: −5.65 to −1.03] and 2.66 vs. 4.81%; MD: −2.15 [−3.23 to −1.08], respectively, *p* -value < 0.01). Similarly, the rate of knee-related readmission was significantly lower in the VRAS TKA cohort (0.69 vs. 1.46%; MD: −0.77 [−1.32 to −0.21]). Although the rate of all-cause readmission was also lower in the VRAS TKA cohort, the difference did not reach statistical significance (1.73 vs. 2.25%; MD: −0.52 [−1.4 to 0.35]).

**Table 4:** Revisit and readmission rates of patients undergoing total knee arthroplasty using either manual approach or VELYS robotic-assisted solution

	VRAS TKA (95% CI)	Manual TKA (95% CI)	Mean difference (95% CI)
Revisit (%)			
Number of patients	866	128,643	–
All-cause	13.86 (11.56 to 16.16)	17.19 (16.99–17.4)	−3.34 (−5.65 to −1.03)
Knee-related	2.66 (1.58–3.73)	4.81 (4.69–4.93)	−2.15 (−3.23 to −1.08)
Readmission (%)			
Number of patients	866	128,643	–
All-cause	1.73 (0.86–2.6)	2.25 (2.17–2.34)	−0.52 (−1.4 to 0.35)
Knee-related	0.69 (0.14–1.25)	1.46 (1.39–1.53)	−0.77 (−1.32 to −0.21)

Abbreviations: CI, confidence interval; TKA, total knee arthroplasty; VRAS, VELYS robotic-assisted solution.

### Secondary Outcomes

Secondary outcomes of the study population including discharge status, operating room time, length of stay, cost of care, and revision rate are presented in Table 5 . The vast majority of patients (96%) in both the VRAS and manual TKA cohorts were discharged to home or home health services. The proportion of patients discharged to skilled nursing facility was also similar in both cohorts (2.1 vs. 2.7%). The VRAS procedures had statistically significant longer operating room time (MD: 4 [2–7] minutes) than manual TKA procedures (138 vs. 134 minutes). The VRAS TKA cohort exhibited a slightly shorter length of stay (3.1 vs. 3.6 days) compared with the manual TKA cohort, although there were only 44 VRAS inpatient cases to begin with. The 90-day revision rate was low and similar for both the VRAS and manual cohorts (0.09 vs. 0.18%).

**Table 5:** Outcomes and resource utilization of patients undergoing total knee arthroplasty using either manual approach or VELYS robotic-assisted solution

	VRAS TKA (95% CI)	Manual TKA (95% CI)	Mean difference (95% CI)
Discharge status			
Number of patients	1,180	161,866	–
Home or home health discharge (%)	96.61 (95.58–97.64)	95.99 (95.89–96.08)	0.62 (−0.41 to 1.66)
Skilled nursing facility discharge (%)	2.12 (1.3–2.94)	2.67 (2.59 to 2.75)	−0.55 (−1.38 to 0.27)
Operating room time	137.96 (135.5–140.42)	133.67 (133.47–133.88)	4.28 (1.81–6.75)
Length of stay (LOS)			
Number of patients	44	16,792	–
Avg. hospital LOS (d)	3.11 (2.73–3.50)	3.63 (3.59–3.68)	−0.52 (−0.91 to −0.13)
90-day cost of care ($)			
Number of patients	866	128,643	–
All-cause ($)	15,357 (14,833–15881)	14944 (14,902–14985)	413 (−112 to 938)
Knee-related ($)	14,955 (14,478–15,433)	14,547 (14,509–14,585)	408 (−69 to 887)
90-day revision rate (%)			
Number of patients	866	128,643	–
Revision rate (%)	0.09 (0.01–0.19)	0.18 (0.09–0.27)	−0.09 (−0.23 to 0.05)

Abbreviations: CI, confidence interval; TKA, total knee arthroplasty; VRAS, VELYS robotic-assisted solution.

#### Cost of Care

The overall 90-day all-cause cost of care was similar for both the VRAS and manual TKA cohorts ($15,357 vs. $14,944; MD: $413 [−$112 to $938]). Similarly, the knee-related 90-day costs were also similar for both cohorts ($14,956 vs. $14,547; MD: $409 [−$69 to $887]). The index costs (i.e., surgical procedure costs) were also similar for both cohorts with supply and operating room costs making about 85% of total cost. On average, VRAS had $6,661 in supply cost compared with $6,459 for manual TKA cases. The operating room costs on average were $6,420 and $5,998 for VRAS and manual TKA cases, respectively.

## Discussion

The objective of the study was to understand the early patient and economic outcomes associated with the use of VRAS in TKA in comparison to manual TKAs. The study identified that both follow-up visits (revisits) and readmission rates were lower for VRAS compared with the manual TKA cohort. All-cause and knee-related revisits occurred at significantly lower rates in the VRAS TKA cohort compared with the manual TKA cohort (19 and 45%, respectively). Furthermore, the VRAS TKA cohort exhibited a statistically significant decrease in knee-related readmissions (53%). These findings are consistent with those found by of Clatworthy, 17 who reported improvements in knee function and pain at early stages with the use of the VRAS technology.

The VRAS TKA cohort exhibited an increase in operating room time (4 minutes, 138 vs. 134 minutes), most likely associated with the integration of robotic instrumentation. Previous studies have reported a learning curve of 5 to 20 cases to achieve surgery times equivalent to the traditional manual approach. 30 31 While the adoption of all new technology requires new skills to be learned and practice to become proficient, this study helps to alleviate the surgeons' concern that adoption of VRAS will be associated with a prolonged learning curve, which will impact their procedure efficiency in the long term as 4-minute difference is not clinically significant.

The economic analysis did not identify significant cost differences between the VRAS and manual TKA cohorts both at index and 90-day cost of care. However, the economic analysis did not account for initial purchase cost for the robotic system. Past studies have reported conflicting findings on cost analysis associated with the use of robotic technologies in TKA. 32 33 34 While increased intraoperative costs were linked to robotic TKA, 33 34 these costs were subsequently compensated by more significant savings in postoperative costs within the 90-day episode of care compared with manual TKA. 34 35 36 The most common reasons for savings included reduced length of stay, decreased opioid prescription, and reduced postdischarge utilization of services associated with the use of robotic TKA. 34 36 37 38

This is the first study to investigate the impact of the VRAS on patient healthcare outcomes and associated costs in a large database. Using a relatively large population across a large geographic area makes the results of this study not only relevant to surgeons, but also to healthcare policy decision-makers and health systems in their effort to provide optimal outcomes and reduced costs. In this regard, our study provides valuable information on the potential benefits and drawbacks of using VRAS compared with manual TKA. Additionally, the use of FSW methodology preserves all patient data, allowing for the inclusion of outlier patients and yielding more representative outcomes and effectively control for confounders between the VRAS and manual TKA cohorts.

The study has several limitations. The Premier Healthcare Database is not specifically designed for research purposes and could answer only limited research questions. It is also prone to issues such as incorrect coding and missing information. Hence, both knee- and all-cause-related outcome numbers were reported. While knee-related outcomes hold greater clinical significance, they may be somewhat underrepresented due to coding errors. All-cause related outcomes comprise all care and potentially can encompass unrelated episodes. The actual rates likely fall between the rates of knee-related and all-cause outcomes. Moreover, the study only included patients from the Premier Healthcare hospitals in the United States and hence may not be reflective of the experience of patients from other hospitals or countries. Additionally, although FSW methodology was used to control confounders between cohorts, unmeasurable variables such as socioeconomic status, surgeon technique, and other factors could still contribute to residual confounding after adjusted analyses. Another limitation is the relatively small cohort of VRAS cases compared with the manual group. Finally, all limitations associated with retrospective observational studies also apply herein.

## Conclusion

Our study presents compelling evidence supporting the benefits of VRAS in TKA, particularly with respect to reduced follow-up revisits and knee-related readmissions. While economic considerations warrant careful examination, our findings suggest that the VRAS has similar hospital costs as manual TKA while not accounting for purchasing fee for the robot.
