# Supplementary material for: Early Clinical and Economic Outcomes for the VELYS Robotic-Assisted Solution Compared with Manual Instrumentation for Total Knee Arthroplasty
Source: J Knee Surg. 2024 Jun 28;37(12):864–72. doi: 10.1055/a-2343-2444 (PMC11405097; doi:10.1055/a-2343-2444)
Supplement: Supplementary file 1 — Supplementary Material [file 10-1055-a-2343-2444-s24apr0070oa.pdf]

Supplementary Table A Elixhauser comorbidities of patients undergoing total knee arthroplasty using either manual approach or VELYS robotic-assisted solution, before and after matching

Variable	Before matching			After matching		
	Manual	VRAS	SMD	Manual	VRAS	SMD
N	128,643	866	–	128,643	866	–
Elixhauser comorbidities (%)						
Congestive heart failure	4.58	2.66	0.103	2.33	2.66	0.021
Cardiac arrhythmia	10.88	8.66	0.075	8.49	8.66	0.006
Valvular disease	2.83	1.85	0.065	2.55	1.85	0.048
Pulmonary circulation disorders	0.75	0.23	0.075	0.45	0.23	0.038
Peripheral vascular disorders	2.54	1.27	0.093	1.56	1.27	0.024
Hypertension uncomplicated	53.79	48.61	0.104	43.40	48.61	0.105
Hypertension complicated	10.57	10.74	0.005	9.47	10.74	0.042
Paralysis	0.04	0.00	0.029	0.01	0.00	0.014
Other neurological disorders	1.90	0.46	0.133	1.26	0.46	0.087
Chronic pulmonary disease	14.73	15.01	0.008	11.64	15.01	0.099
Diabetes uncomplicated	14.83	9.93	0.149	11.17	9.93	0.04
Diabetes complicated	7.00	7.27	0.011	3.86	7.27	0.15
Hypothyroidism	15.07	15.70	0.018	14.21	15.70	0.042
Renal failure	8.33	9.01	0.024	8.53	9.01	0.017
Liver disease	1.50	1.15	0.03	1.22	1.15	0.006
Peptic ulcer disease excluding bleeding	0.20	0.12	0.022	0.23	0.12	0.027
AIDS/HIV	0.06	0.00	0.033	0.04	0.00	0.028
Lymphoma	0.15	0.12	0.011	0.07	0.12	0.016
Metastatic cancer	0.00	0.00	<0.001	0.00	0.00	<0.001
Solid tumor without metastasis	0.15	0.12	0.01	0.11	0.12	0.002
rheumatoid arthritis/collagen	3.94	5.31	0.065	3.34	5.31	0.097
Coagulopathy	1.82	1.15	0.055	2.54	1.15	0.103
Obesity	32.14	27.14	0.11	26.80	27.14	0.008
Weight loss	0.07	0.00	0.037	0.02	0.00	0.021
Fluid and electrolyte disorders	2.43	1.96	0.032	2.15	1.96	0.013
Blood loss anemia	0.16	0.12	0.011	0.07	0.12	0.015
Deficiency anemia	1.21	1.62	0.035	1.15	1.62	0.04
Alcohol abuse	0.52	0.58	0.008	0.24	0.58	0.053
Drug abuse	0.79	1.15	0.038	0.57	1.15	0.063
Psychoses	0.22	0.12	0.025	0.13	0.12	0.004
Depression	10.49	11.66	0.037	10.19	11.66	0.047

Abbreviations: SMD, standardized mean difference; TKA, total knee arthroplasty; VRAS, VELYS robotic-assisted solution.

 © 2024. The Author(s).

Supplemental Table B Functional comorbidities of patients undergoing total knee arthroplasty using either manual approach or VELYS robotic-assisted solution, before and after matching

Variable	Before matching			After matching		
	Manual	VRAS	SMD	Manual	VRAS	SMD
N	128,643	866		128,643	866	
Functional comorbidities (%)						
FCI 01 arthritis	97.93	99.65	0.158	99.48	99.65	0.021
FCI 02 osteoporosis	3.24	0.92	0.163	2.04	0.92	0.092
FCI 03 asthma	8.76	10.51	0.059	7.31	10.51	0.113
FCI 04 COPD	6.12	4.97	0.051	4.47	4.97	0.023
FCI 05 angina	0.07	0.00	0.036	0.06	0.00	0.035
FCI 06 CHF or heart disease	63.53	55.77	0.159	52.9	55.77	0.063
FCI 07 heart attack	2.99	1.73	0.083	1.92	1.73	0.014
FCI 08 neurological disease	32.63	45.03	0.257	31.06	45.03	0.291
FCI 09 stroke or TIA	0.06	0.00	0.035	0.18	0.00	0.061
FCI 10 diabetes	21.73	17.09	0.118	15.71	17.09	0.042
FCI 11 PVD	1.10	0.35	0.089	0.56	0.35	0.032
FCI 12 upper GI disease	28.03	24.25	0.086	24.85	24.25	0.014
FCI 13 depression	10.35	11.66	0.042	10.09	11.66	0.05
FCI 14 anxiety or panic disorders	11.35	13.05	0.052	10.98	13.05	0.064
FCI 15 visual impairment	0.38	0.12	0.053	0.20	0.12	0.021
FCI 16 hearing impairment	1.51	0.58	0.092	1.02	0.58	0.05
FCI 17 degenerative disc disease	0.83	0.23	0.083	0.70	0.23	0.069
FCI 18 obesity	32.14	27.14	0.11	28.58	27.14	0.032

Abbreviations: CHF, congestive heart failure; COPD, chronic obstructive pulmonary disease; FCI, Functional Comorbidity Index; GI, gastrointestinal; PVD, peripheral vascular disease; SMD, standardized mean difference; TKA, total knee arthroplasty; VRAS, VELYS robotic-assisted solution.

 © 2024. The Author(s).
